# Metastasectomy of Solitary Adrenal Metastasis From Small Cell Lung Cancer

**DOI:** 10.1177/2324709617740909

**Published:** 2017-11-03

**Authors:** Dat Cao Pham, Ziad Awad, Bradford S. Hoppe, Jason Hew, Ke Ning

**Affiliations:** 1 UF Health Jacksonville, FL, USA

**Keywords:** small cell lung cancer, solitary adrenal gland metastasis, adrenalectomy, metastasectomy, treatment

## Abstract

Most extensive-stage small cell lung cancers have disseminated disease at diagnosis, and solitary adrenal metastasis from small cell lung cancer is rare. We report the case of a 51-year-old man with extensive-stage small cell lung cancer with solitary right adrenal metastasis (T2N0M1), who was cured with resection of primary tumor, chemotherapy (cisplatin, etoposide), adrenalectomy, consolidative thoracic radiotherapy, and prophylactic cranial irradiation. He remained cancer free, 7.5 years since diagnosis. For small cell lung cancer with solitary adrenal gland metastasis, metastasectomy combined with chemotherapy and radiotherapy can be curative and enable long-term survival. This treatment approach should be further studied, and similar cases should be reported.

## Background

Small cell lung cancer accounts for about 15% of all lung cancers. Although it is sensitive to chemotherapy and radiotherapy, it carries a poorer prognosis compared with non–small cell lung cancer due to its aggressive clinical course, early hematogenous metastasis, rapid doubling time, and high growth fraction. Most patients with extensive-stage have disseminated disease on diagnosis. In extensive-stage small cell lung cancer, solitary metastasis to the adrenal gland is rare and the role of adrenalectomy has not been studied. We report the case of a man with extensive-stage small cell lung cancer with solitary metastasis to the right adrenal gland, who was cured with resection of primary tumor, chemotherapy, adrenalectomy, and consolidative thoracic radiotherapy.

## Case

**Figure 1.**
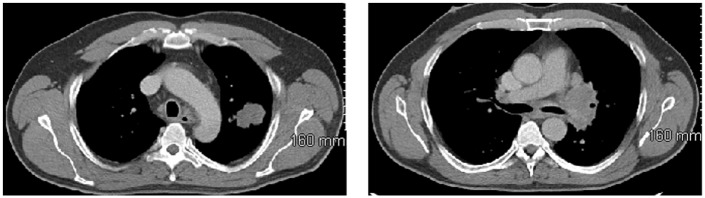
CT chest showing a left upper lobe mass and left mediastinal lymphadenopathy.

**Figure 2.**
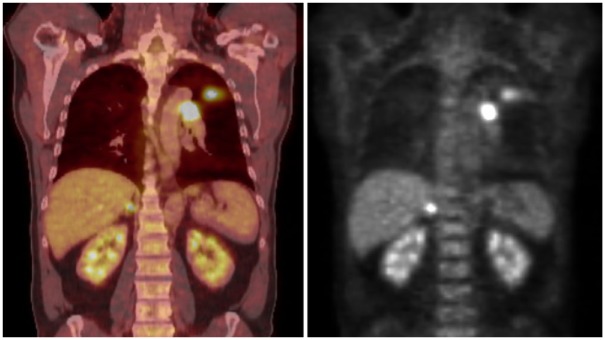
PET-CT showing hypermetabolic left upper lobe mass, left mediastinal lymphadenopathy, and a mass in right adrenal gland.

A 51-year-old man presented with shortness of breath and left-sided chest pain in March 2010. He was a heavy smoker. Radiographs and computed tomography (CT) scan of the chest showed a left upper lobe mass measuring 3.6 cm × 2.5 cm × 3.2 cm and enlarged left mediastinal lymph nodes (Figure 1). Positron emission tomography (PET) scan showed a hypermetabolic left upper lobe mass with standardized uptake value (SUV) of 19.2, extensive left hypermetabolic mediastinal lymph nodes with SUV of 7.5, and a 1-cm mass in the right adrenal gland with SUV of 6.8, concerning for metastasis (Figure 2). Transbronchial biopsies of the mediastinal lymph nodes were negative for malignancy. Subsequent mediastinoscopic biopsies of these lymph nodes were also negative and only showed reactive tissue with abundant lymphocytes. Video-assisted thoracoscopy showed large lymph nodes in the anterior left hilum, which were excised. Surprisingly, these lymph nodes also showed similar reactive tissue with many lymphocytes. The patient then underwent wedge resection of the left upper lobe mass, which revealed small cell carcinoma that was strongly positive for p16, cytokeratin, synaptophysin, and chromogranin and negative for TTF-1, p63, CK5/6, and high-molecular-weight keratin. There were extensive tumor necrosis, lymphovascular invasion, and presence of infiltrating lymphocytes. Chemotherapy with cisplatin, etoposide, and pegfilgrastim support was started and definitive staging and potentially curative therapy with right adrenalectomy was planned. After completing 3 cycles of chemotherapy, he underwent right adrenalectomy. The resected adrenal mass confirmed metastatic small cell lung cancer, without infiltrating lymphocytes. Pathologic stage was T2N0M1. He underwent additional chemotherapy with cisplatin and etoposide. PET scan after 6 cycles of chemotherapy showed a slight residual uptake in the left hilar area. He received 2 additional cycles of chemotherapy, completing a total of 8 cycles in January 2011. He then received prophylactic cranial irradiation and consolidative radiotherapy to the thorax. PET-CT scans in 2012 and 2014 and CT chest in 2016 showed no evidence of recurrence. At the most recent visit in 2017, the patient was doing well, and remained cancer free, 7.5 years since diagnosis.

## Discussion

Standard treatment for extensive-stage small cell lung cancer is mainly palliative chemotherapy with a platinum compound plus etoposide or irinotecan. Two-year overall survival rate is less than 5% for extensive-stage small cell lung cancer. Although resection of the primary and solitary adrenal metastatic lesion can improve survival and can be curative in non–small cell lung cancer,^1^ the benefit of adrenalectomy of solitary adrenal metastasis in small cell lung cancer has not been well studied, perhaps due to the rarity of cases. Pant-Purohit et al reported of a patient with limited-stage small cell lung cancer, who developed a solitary metastasis to the adrenal gland 5 years after diagnosis and was successfully treated with left adrenalectomy with a disease-free survival of more than 66 months since adrenalectomy.^2^ Our patient differs in that he presented with extensive-stage small cell lung cancer with solitary adrenal metastasis. Fortunately, the primary tumor was resectable, and all lymph nodes though hypermetabolic were surprisingly just reactive, likely due to immunologic responses to cancer. After wedge resection of primary, and 3 cycles of chemotherapy, right adrenalectomy was performed for staging and treatment of solitary metastasis. After completion of chemotherapy, he received prophylactic cranial irradiation and consolidative thoracic radiotherapy since he only had wedge resection of the primary tumor. Our case demonstrated that metastasectomy of solitary adrenal metastasis from small cell lung cancer in combination with primary tumor resection, chemotherapy, and radiotherapy can be curative and enable long-term survival. This treatment approach should be further investigated in carefully selected patients, and similar cases should be reported.
